# Artificial Intelligence as a Tool for Self-Care in Patients with Type 1 and Type 2 Diabetes—An Integrative Literature Review

**DOI:** 10.3390/healthcare13080950

**Published:** 2025-04-21

**Authors:** Vera Persson, Ulrica Lovén Wickman

**Affiliations:** 1 Department of Region Halland, 301 80 Halmstad, Sweden; vera.persson@regionhalland.se; 2 Department of Health and Caring Sciences, Linnaeus University, 391 82 Kalmar, Sweden

**Keywords:** artificial intelligence, diabetes mellitus, district nurse, machine intelligence, nurse’s role, review

## Abstract

**Background/Objectives**: Diabetes is a common public health disease that affects patients mentally, physically, and economically. It requires lifestyle changes such as blood sugar control and regular contact with healthcare services. Artificial intelligence has developed rapidly in many different areas in recent years, including healthcare and nursing. The aim of this study is to explore how artificial intelligence can be used as a tool for patients with diabetes mellitus. **Methods**: An integrative literature review design was chosen according to Whittemore and Knafl (2005). Electronic searches in databases were conducted across Pub-Med, CINAHL Complete (EBSCO), and ACM Digital Library until September 2024. A total set of quantitative and qualitative articles (n = 15) was selected and reviewed using a Mixed Method Appraisal Tool. **Results**: Artificial intelligence is an effective tool for patients with diabetes mellitus, and various models are used. Three themes emerged: artificial intelligence as a tool for blood sugar monitoring for patients with diabetes mellitus, artificial intelligence as a decision support for diabetic wounds and complications, and patients’ requests for artificial intelligence capabilities in relation to tools. Artificial intelligence can create better conditions for patient self-care. **Conclusions**: Artificial intelligence is a valuable tool for patients with diabetes mellitus and enables the district nurse to focus more on person-centered care. The technology facilitates the patient’s blood sugar monitoring. However, more research is needed to ensure the safety of AI technology, the protection of patient privacy, and clarification of laws and regulations within diabetes care.

## 1. Background

Patients with diabetes face challenges related to diet, exercise, and medication and require continuous monitoring of their blood sugar levels. These demands can negatively affect the patient’s quality of life [1]. Patients need support in managing their disease. In the advanced nurse-district nurse role, patients with diabetes are regularly met to provide support, encouragement, advice, and education. This way, the patient receives support to accept their disease, manage their medication, and develop healthy habits regarding diet and exercise. It strengthens the patient’s ability to manage their diabetes [2].

Diabetes is a metabolic disease that causes hyperglycemia, which means elevated glucose concentration [3]. An autoimmune reaction causes type 1 diabetes. The exact trigger for this is unknown, but it might be linked to infections, diet, stress, or environmental factors. The treatment is to supply the body with insulin to lower glucose levels and avoid complications [3,4,5]. In type 2 diabetes, the number of beta cells decreases, which results in reduced insulin production and increased insulin resistance, primarily in the liver, skeletal muscles, and adipose tissue. In type 2 diabetes, the body produces an insufficient amount of insulin. When insulin secretion is impaired, the glucose level in the blood rises [5]. Risk factors for developing type 2 diabetes include genetic predisposition, unhealthy lifestyle habits, lack of exercise, smoking, and alcohol. Other risk factors are advanced age, inflammatory conditions, pregnancy, and high blood pressure [4]. Type 1 and type 2 diabetes affect several organs in the body and can lead to complications associated with the disease. Complications that may arise include cardiovascular diseases and metabolic disorders such as obesity, high blood pressure, lipid metabolism disorders, and impaired kidney function. Type 1 and type 2 diabetes can also lead to microvascular complications such as neuropathy, nephropathy, and retinopathy [4,6,7]. Treatment of patients with type 2 diabetes often involves dietary changes, physical activity, and medication [8]. It is also important to regularly monitor blood sugar levels and inspect feet daily to avoid complications such as diabetic neuropathy and diabetic foot ulcers, which can lead to foot amputation if not treated in time [9,10,11].

The patient’s perspective of living with diabetes entails both psychological and physical consequences for the patient [12]. The disease involves a lifestyle change with continuous monitoring and regulation of blood sugar, medication adjustments, and improved diet and exercise habits [1]. Some of the challenges are time-consuming. It can cause emotional, social, and psychological strain for the patient and the family. It can become costly with the purchase of medications and regular doctor visits, and in the long run, it can mean a significant financial challenge [13,14]. Another strain for patients with type 1 diabetes is that 50% of hypoglycemia episodes occur at night. It is a challenge for the patient to monitor their blood sugar at night with traditional methods [15]. The various strains can lead to anxiety, worry, and depressive symptoms that can negatively affect the patient in the workplace and can impair quality of life and function [13,14]. The application of artificial intelligence (AI) is shown in one study, which found factors associated with poor self-management activities among patients with type 2 diabetes. The use of a wide range of neurons in the hidden layers to train the artificial neural network models improved outcomes, confirming the model’s effectiveness and efficiency in assessing diabetes self-care activities [16]. Another study expresses that AI applications have the potential to transform diabetes care and help patients achieve better blood glucose control, reduce hypoglycemic episodes, and reduce complications. AI applications offer accuracy, efficiency, ease of use, and satisfaction for patients, families, and healthcare professionals [17]. Limitations of AI are expressed as human factors, technical factors, and barriers including cost, access, implementation, and limitations of data. Other barrier concerns are about security and data protection [18]. The research also refers to ethical and legal aspects of protecting the patient [19].

According to Swedish law, self-care is defined as a health and medical measure where healthcare professionals have assessed that the patient can independently act to investigate, prevent, and treat disease (SFS 2022:1250) [20]. A middle-range theory conceptualizes self-care as an integrated and dynamic process requiring commitment and interaction between the individual and their environment to include a range of chronic illnesses, like diabetes mellitus. The theory outlines three fundamental dimensions of self-care: self-care maintenance, self-care monitoring, and self-care management [21]. These dimensions collectively describe how individuals sustain their health through proactive behaviors, monitor changes in symptoms or conditions, and manage emerging health issues through appropriate self-care actions [21]. Self-care is important in both type 1 and type 2 diabetes and primarily involves glucose measurement and regulation of glucose levels. Self-care in diabetes means following recommendations to prevent complications and improve quality of life by promoting better blood sugar control, where HbA1c is a measure to assess blood sugar over time [22]. There are instruments available to measure self-care ability in patients with diabetes. The most used instruments are the Self-Care Activities (SDSCA) and the Self-Care Inventory-Revised (SCI-R) [23,24]. A study tested the Self-Care of Diabetes Inventory (SCODI), based on the theory of self-care as a continuous process to maintain health and manage disease through health-promoting methods [21]. The theory includes eating healthily, engaging in physical activity, and taking medications as prescribed. The theory also describes how the patient monitors their self-care by regularly observing symptoms and being attentive to how the body reacts. Another important aspect is that the patient manages health problems that arise, such as contacting healthcare in case of deviations, adjusting treatment, and changing lifestyle. It is also important that the patient believes in their self-care ability to perform health-promoting actions that are crucial for a successful self-care process despite obstacles and challenges [25].

Barriers and individual preferences should be considered to motivate patients to improve their self-care ability effectively. Self-care diagnostics can identify barriers to the patient’s self-care and determine the need for further education. It includes gathering information about the patient’s knowledge of diabetes and its treatment and their resources and attitudes, such as dietary habits, exercise habits, and cultural background [25]. Education gives the patient knowledge about their disease and is an important part of preventing complications and increasing self-care to achieve a better quality of life [26].

AI is the science and technology used to create an intelligent machine. AI works and behaves like a human and can solve tasks like a human by using visual perception, speech recognition, decision-making, and translation between different languages [27]. Progress has been made in introducing AI to medicine. AI is used clinically to effectively predict, diagnose, assess, and treat diseases. This is because AI can analyze and manage unlimited data more efficiently than the human brain is capable of, and AI also has a better ability to predict outcomes [27,28,29,30]. AI can be integrated into information systems to increase efficiency and accuracy in healthcare. By analyzing patient data, AI can also predict complications and contribute to more personalized treatment plans, promoting person-centered care [31]. AI is a tool that can create better conditions for patient self-care and help facilitate the work of district nurses. AI has recently developed rapidly in many areas, including healthcare and nursing [32].

Living with diabetes affects patients psychologically, physically, and economically. Managing diabetes involves lifestyle changes, including medication intake, dietary and exercise adjustments, blood sugar monitoring, and regular healthcare interactions. With the rapid development of AI technology, there is a growing need to integrate AI into diabetes care. AI can streamline diabetes management by assisting patients with diabetes mellitus. The knowledge gap is to see how district nurses can use AI tools to help patients monitor their blood sugar levels, identifying suitable AI tools for diabetes care and understanding their benefits for patients. Increased knowledge and information are essential for effectively using AI technology. Therefore, exploring how patients with diabetes can utilize AI for support in their treatment and care is of interest.

## 2. Methods

### 2.1. Design

The chosen design was an integrative literature review that combined quantitative and qualitative research and involved the integration of various data sources. According to the method for an integrative review, identifying a problem is followed by data collection and data analysis. Data collection can be extensive and broad, so it was important to narrow it by describing a clear purpose [33].

### 2.2. Eligible Criteria and Data Collection

**Figure 1:**
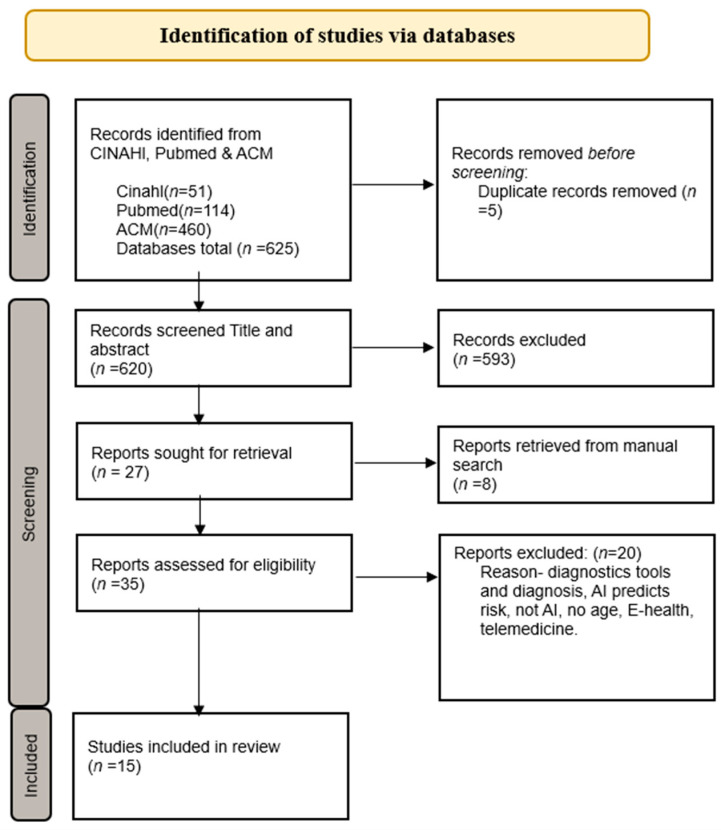
PRISMA flowchart [35].

The search keywords and their combinations were performed closely with a librarian. According to the PEO model, the database of thesauruses and Medical Subject Headings (MeSH) terms that generated synonyms were used to identify relevant search terms for the preliminary search. Electronic searches were conducted across Pub-Med, CINAHL Complete (EBSCO), and the ACM Digital Library until September 2024. The inclusion criteria in the articles were patients over 18 years old and those with type 1 or 2 diabetes. The inclusion criteria for selecting articles and their research method were qualitative and quantitative original peer-reviewed articles. Exclusion criteria were children, pregnant women, and other types of diabetes, such as prediabetes and diabetes insipidus. Exclusion criteria for the scientific articles were being older than ten years and not written in English. The study included empirical studies that could be credible sources. The inclusion and exclusion criteria of articles increase the study’s reproducibility [34]. In an integrative review, the primary sources should be of high quality by adhering to inclusion and exclusion criteria to specify their area in the study [33]. Manual searching can be used when data search does not provide sufficient articles [34]. The search terms reported were artificial intelligence, diabetes mellitus, digital health technology, district nurse, and patient-centered care. The search terms, Prisma flow diagram, and article matrix summarize the search process (Figure 1, Table 1, Appendix A).

### 2.3. Quality Appraisal

The articles were quality-reviewed using the Mixed Methods Appraisal Tool (MMAT, version 2018) [50]. The quality review was a fundamental basis for this integrative overview. It was crucial to ensure that the conclusions were reliable and maintained a high-quality scientific standard [33]. It was complex to evaluate the quality of primary sources because the integrative review included articles with varying research designs and methods, making the process challenging. There is no gold standard for assessing quality since the quality could vary depending on the research design in the articles. The quality appraisal score of the articles is presented in Table 1. Both authors were involved in screening, quality appraisal, data extraction, and data analysis.

### 2.4. Data Analysis

The analysis followed the five steps described by [33]: (1) data reduction, (2) data display, (3) data comparison, (4) conclusions, and (5) verification. According to the first step of the analysis, the focus was on data reduction to extract relevant information from each article. This included parts of the results that answered the purpose. Meaning units, which were specific sentences and parts of the text directly related to the research question, were extracted. Meaning units were summarized to make the information clearer and easier to handle. After data reduction and data display, a data comparison was made, where the extracted data from different sources were compared to identify patterns, similarities, and differences. By comparing the results from different articles, the authors could identify common categories that recurred in the material. The data display was presented in a table for comparison and interpreted [33] (Table 1). To clearly present and organize the extracted information, tables and figures were used, which helped to separate and provide an overview of the different meaning units and how they related to each other. In this way, data could be presented in a structured and clear manner, facilitating further analysis.

### 2.5. Ethical Considerations

In a literature review, no ethics application is required as the analysis is conducted on already published articles. The benefit of this study is that the technology is relatively unexplored, while it is developing rapidly. Therefore, it is important to gather knowledge to improve diagnostics and treatment, increase efficiency, and improve patient safety by investigating the role and use of artificial intelligence in healthcare. The study can benefit patients with diabetes by highlighting and using AI tools that effectively analyze and interpret large amounts of data and support the district nurse’s care work. To improve the quality of care in an ethical and responsible manner, it is important to consider respect for human dignity, although this is outside the scope of this study. It is important to ensure that AI tools consider patient privacy and autonomy by protecting patient data through data protection measures. AI also needs to be technically safe and robust for secure use. The risks associated with AI technology can be avoided through safe design, regulation of AI, and safe use [51].

## 3. Results

The reviewed studies were conducted in China (n = 5), the United States (n = 4), United Kingdom (n = 2) and single studies were conducted in Russia, Singapore, Taiwan, and Turkey (Table 1). The results are presented as three themes (Table 2).

### 3.1. Artificial Intelligence as a Tool for Blood Sugar Monitoring for Patients with Diabetes Mellitus

The results show that AI XGBoost, multilayer perception (MLP), REFS (Reverse Engineering and Forward Simulation), and Random Forest (RF) can be used as tools for patients with diabetes. AI can be used to monitor blood sugar levels and predict complications [11,37,38,40,42,43,45,46,47,48]. Patients with type 2 diabetes fasted during Ramadan and were examined with five different machine learning techniques. The goal was to study which machine learning technique was best at predicting hyperglycemia and hypoglycemia. The results showed that the XGBoost model was best at predicting blood sugar levels. XGBoost was better at predicting the risk of hyperglycemia. XGBoost is a machine learning algorithm that efficiently processes information and uses input factors to predict an outcome [37]. Another result showed that the introduction of the machine learning model multilayer perception (MLP) can be used to predict hyperglycemic crises in patients. MLP learns to predict hyperglycemic crises by using a network that collects and processes information to predict outcomes. It is possible to get a real-time risk assessment of hyperglycemia by pressing a button [38]. It showed that algorithms such as deep learning (DL) and machine learning (ML) could predict blood sugar levels. The MLP model was best at predicting hypoglycemia in patients with type 1 diabetes at night [40].

Another study highlighted the machine learning model REFS (Reverse Engineering and Forward Simulation), which could predict risk factors causing hypoglycemia. REFS analyzed patient information over a 12-month period. Relevant patient information included HbA1c, previous episodes of hypoglycemia, insulin use, and whether the patient had been hospitalized related to diabetes. Based on this information, patients at risk of hypoglycemia were identified [42]. Random forest (RF) has higher complexity and performs better than decision processes. RF captures multiple factors and is more accurate. The decision process uses two factors to predict hypoglycemia, making it easier to use and interpret. The decision process had an accuracy of 80%, while RF achieved an accuracy of 87% in predicting the risk of hypoglycemia during exercise in adults with type 1 diabetes [43]. The results also showed that the machine learning model XGBoost performed best at predicting the risk of hypoglycemia in hospitalized patients with diabetes. To predict the outcome, XGBoost uses a decision process [45]. Another result showed that one of the machine learning models (ML), Extreme Gradient Boosting (XGBoost), predicted the risk of severe hypoglycemia in patients with diabetes [52] The results also showed that the machine learning methods ensemble learning and Extreme Gradient Boosting (XGBoost) were the most effective at predicting fasting blood sugar and HbA1c in patients with type 2 diabetes. XGBoost can handle a large amount of data. Ensemble learning is an advanced machine learning model and works meticulously [47]. Another result showed that XGBoost3 performed best in predicting hypoglycemia in patients with type 2 diabetes. XGBoost3 is a machine learning algorithm that uses patient information to analyze and process the information to predict the risk of hypoglycemia [10]. It showed that the Treatment Pathway Graph-Based Estimation (TPGE) model is best at predicting HbA1c levels in patients with type 2 diabetes. Additionally, it can predict how different treatment options will affect HbA1c. The TPGE model analyzed treatments and calculated the probability of how effective each treatment would be [48].

### 3.2. Artificial Intelligence as a Decision Support for Diabetic Wounds and Complications

AI technology as C4W, Extreme Gradient Boosting (XGBoost) and random forest (RF) can be used as a decision support system for risk assessment of wounds and amputations in patients [36,41,49]. C4W has good reliability in assessing wound images regarding length, width, and area. C4W was compared with traditional wound measurement and tended to overestimate wound size. C4W stands for CARES4WOUNDS, an artificial wound imaging system. The system is designed to automatically analyze and measure diabetic wounds [36]. It showed that the AI tool recommends the same amputation level as the surgeons in 83.3% of the studied patient cases. The AI tool used to evaluate amputation levels was ChatGPT-4.0. The tool was chosen because it did not require any training data set unlike other machine learning models [41]. Risk prediction models were used to predict amputations in patients with diabetic foot ulcers grade 3. The results showed that XGBoost and RF were the best at predicting diabetic foot ulcers grade 3. However, XGBoost was the best of the two. XGBoost works accurately and has the best predictive ability. RF uses a decision process that processes complex patient information to provide a reliable result [49].

### 3.3. Patients’ Requests for Artificial Intelligence Capabilities in Relation to Tools

Patients have expressed a desire for education and information to be able to use AI technology [39,44]. The results showed that patients have the following needs: information, safety, and trust in AI. Patients wanted access to important information and education regarding AI. The requests are to receive education through brochures, on-site training, digital support, and access to other patient experiences to understand how to handle warnings and notifications from AI technology. This is to use AI in a safe and secure manner and build trust [44]. Patients who have moved away from home and started university describe their support from the technology that provides remote monitoring of blood sugar and sends notifications via app and sensor with information about blood sugar levels to people around them. Notifications went to roommates or friends to support the patient with type 1 diabetes. It is shown to result in the patient gaining increased independence, which is positive, and a sense of security in managing their blood sugar with reminders to those around them, so that patients with diabetes take actions such as eating or taking insulin [39].

## 4. Discussion

The purpose of this study was to explore how AI can be used as a tool for patients with diabetes mellitus. The results showed that several suitable tools could be beneficial for patients with diabetes mellitus and AI can be used to monitor blood sugar levels.

AI as a Tool for Blood Sugar Monitoring showed that the machine learning model XGBoost performed best in predicting the risk of hypoglycemia in patients with diabetes. Several machine learning models have been used to predict hypoglycemia in previous research. One study described how continuous glucose monitoring (CGM) has developed. AI can be used for the next generation of CGM systems to predict hypo- and hyperglycemia [53]. CGM provides continuous information that cannot be achieved with single blood tests, making it an important tool in diabetes care [54]. Another study shows that patients with type 2 diabetes tend to check their blood sugar too infrequently, increasing the risk of hypoglycemia. The machine learning models Support Vector Machine (SVM) and Random Forest (RF) have also been shown to predict hypoglycemia with high accuracy [55]. In diabetes care, the primary goal is to maintain stable glucose levels, and AI technology facilitates this by analyzing patient data and identifying deviations in glucose levels. CGM systems are a clear example of how AI has been implemented in diabetes care [28,56,57]. AI also promotes patient autonomy and makes them more involved in their care, increasing their ability to make informed decisions about their treatment. District nurses play an important role in informing patients about the benefits and risks of AI tools to minimize the risk of hypoglycemia.

The results showed that MLP was best at predicting hypoglycemia in patients with type 1 diabetes at night. According to [53], nocturnal hypoglycemia occurs in diabetics. New technology such as continuous glucose monitoring (CGM) can reduce episodes of hypoglycemia. Furthermore, it is described how CGM systems use various machine learning models to predict nocturnal hypoglycemia. Another study has shown that MLP is effective in predicting and warning patients with type 1 diabetes of hypoglycemia during the night. Warning systems are important to prevent life-threatening episodes of hypoglycemia [15]. District nurses have a central role in integrating technology into healthcare. According to [58], district nurses can start from the patient’s life story, which helps to create a partnership between the caregiver and the patient. It is important that district nurses work based on person-centered care, and when machine learning models are used, they can support an individualized care plan based on the patient’s life situation. It is important to work according to person-centered care, where the caregiver builds relationships with the patient and integrates them into their care. Building a relationship based on mutual respect is crucial to creating trust between the patient and the district nurse. Another important aspect is educating the patient on how CGM works and how to interpret analyses from machine learning [59].

AI technology was shown to be used as a decision support system for risk assessment of wounds and amputations in patients. CARES4WOUNDS (C4W), an artificial wound imaging system, has good reliability in assessing wound images regarding length, width, and area. C4W was compared with traditional wound measurement and tended to overestimate wound size. It showed that the AI tool recommends the same amputation level as the surgeons in 83.3% of the studied patient cases. The AI tool used to evaluate amputation levels was ChatGPT-4.0. The tool was chosen because it did not require any training data set unlike other machine learning models [41]. Risk prediction models were used to predict amputations in patients with diabetic foot ulcers grade 3. The results showed that Extreme Gradient Boosting (XGBoost) and random forest (RF) were the best at predicting diabetic foot ulcers. However, XGBoost was the best of the two. XGBoost works accurately and has the best predictive ability. Random Forest (RF) uses a decision process that processes complex patient information to provide a reliable result [49].

Patients expressed requests for AI capabilities in relation to tools. Patients expressed a desire for education and information to be able to use AI technology. The results showed that patients have several needs: information, safety, and trust in AI. Patients wanted access to important information and education regarding AI. The requests are to receive education through brochures, on-site training, digital support, and access to other patient experiences to understand how to handle warnings and notifications from AI technology. This is to use AI in a safe and secure manner and build trust [44]. The use of AI in diabetes management involves continuous monitoring. Ensuring that this data is used ethically and that patients’ privacy is maintained is critical. This requires a multi-faceted approach involving ethical guidelines, patient education, transparent AI systems, and careful integration into clinical practice [60]. AI tools for diabetes management, such as predictive models for blood glucose levels, can exhibit bias if the training data is not diverse. This can lead to less accurate predictions for specific populations, potentially impacting the effectiveness of diabetes management. Tools like continuous glucose monitors require active patient engagement. Patients need to input data accurately, which can be challenging [61].

Patients who have moved away from home and started university describe their support from technology that provides remote monitoring of blood sugar and sends notifications via app and sensor with information about blood sugar levels to people around them. Notifications went to roommates or friends to support the patient with type 1 diabetes. It is shown to result in the patient gaining increased independence, which is positive, and a sense of security in managing their blood sugar with reminders to those around them, so that patients with diabetes take actions such as eating or taking insulin [39].

### Strengths and Limitations

The strength of an integrative literature study is that it combines both quantitative and qualitative articles, which increase transferability and provide a broader understanding of the research problem. The data presented are based on the MMAT quality review score, which makes the results neutral and traceable. The review of primary sources in integrative studies should be of high quality and based on inclusion and exclusion criteria to specify its area. The strength is that it increases the study’s credibility, reproducibility, and transferability. Block searching was performed and was designed with the support of a librarian. The limitation is that there are differences in subject terms and free text terms, which can make the search broad or stripped down depending on which database is used. This can lead to misleading results and conclusions. To minimize that risk, a librarian was helpful. The limitations lie in the limited research on AI as a tool for patients with diabetes. Most articles focus on how AI can predict the risk of developing diabetes, diagnose a condition, or support e-health. Some articles did not specify age in their method and were therefore excluded.

## 5. Conclusions

The major finding is that AI is used as a suitable tool and is beneficial for patients with diabetes mellitus. Technology can predict complications, monitor blood sugar, and function as a decision support system. AI technology promotes self-care ability in patients with diabetes and improves quality of life. This can benefit healthcare professionals in their decision-making process. It also emerged that patients want more information and education on using AI technology, which can provide safe and secure use of AI technology. The district nurse should work based on person-centered care, having the patient and their life story at the center. AI technology is considered to support the district nurse in their work and can assist the patient in self-care.

## 6. Clinical Implications

The use of AI technology in diabetes care has several positive effects. AI can analyze large amounts of data in real time, helping healthcare professionals make better decisions and reduce the risk of complications both in the short and long term. AI can also relieve district nurses from administrative tasks, such as monitoring blood sugar levels and patient data, giving them more time to engage in person-centered work and reduce their workload and stress. Another advantage is that AI can provide medication and lifestyle advice recommendations and improve patient care. At the same time, there must be a clear strategy for handling technical errors and interruptions so that patient data is stored securely. Despite the advantages, there are challenges. AI technology can create insecurity and a lack of trust among patients, especially if they worry about the safety and privacy of their data. This can lead to patients not starting or completing treatment. Additionally, too much reliance on AI can make patients passive, something that can be counteracted through education. There are also risks for district nurses. If AI makes incorrect assessments or recommendations, it can lead to a deficient care plan, and the question of responsibility for such mistakes is not always clear. It is important that healthcare professionals do not forget that AI is only a support tool and does not replace their own clinical judgments, which can create dilemmas when their assessments do not align with AI.

## Figures and Tables

**Table 1:** Article matrix.

Author(s)/Title Year, Country	Title	Method	Artificial Intelligence	Quality Score
1. Chan et al. (2022) [36] Singapore	Clinical validation of an artificial intelligence-enabled wound imaging mobile application in diabetic foot ulcers	Prospective cross-sectional study	Artificial intelligence-enabled wound imaging mobile application (CARES4WOUNDS [C4W]-system	High
2. Elhadd et al. (2020) [37] United States	Artificial Intelligence (AI) based machine learning models predict glucose variability and hypoglycaemia risk in patients with type 2 diabetes. on a multiple drug regimen who fast during ramadan	Prospective observational cohort study	XGBoost, a machine learning algorithm for AI, achieves high predictive performance for normal and hyperglycemic deviations	High
3. Hsu et al. (2023). [38] Taiwan	Using artificial intelligence to predict adverse outcomes in emergency department patients with hyperglycemic crises in real time	Quantitative	MLP to predict negative outcomes of hyperglycemic crises in real-time	High
4. James, S. et al. (2023) [39] United Kingdom	Chronic Care in a Life Transition: Challenges and Opportunities for Artificial Intelligence to Support Young Adults with Type 1 Diabetes Moving to University.	Qualitative interview study	Patients with type 1 diabetes who move out and start university described their support from technology	High
5. Kozinetz, R.M. et al. (2024) [40] Russia	Machine Learning and Deep Learning Models for Nocturnal High- and Low-Glucose Prediction in Adults with Type 1	Quantitative	MLP predicts hypoglycemia in patients with type 1 diabetes at night	High
6. Mert et al. (2024) [41] Turkey	Artificial intelligence’s suggestions for level of amputation in diabetic foot ulcers are highly correlated with those of clinicians, only with exception of hindfoot amputations	Quantitative	The AI tool ChatGPT-4.0 provides recommendations for amputation.	High
7. Mueller, L. et al. (2020) [42] United States	Application of Machine Learning Models to Evaluate Hypoglycemia Risk in Type 2 Diabetes.	Quantitative	Application of Machine Learning Models	High
8. Reddy, R. et al. (2019) [43] United States	Prediction of Hypoglycemia During Aerobic Exercise in Adults with Type 1 Diabetes	Randomized clinical study, Quantitative	Machine learning, RF model, predicts hypoglycemia during exercise in patients with type 1 diabetes	High
9. Robinson, R. et al. (2023) [44] United States	Artificial Intelligence in Health Care-Understanding Patient Information Needs and Designing Comprehensible Transparency	Qualitative study	Preferences for education and information when using AI	High
10. Ruan et al. (2020) [45] United Kingdom	Predicting the Risk of Inpatient Hypoglycemia with Machine	Retrospective cohort study, Quantitative	The machine learning model with the best performance was the XGBoost model, which predicts the risk of hypoglycemia	High
11. Shi et al. (2024) [46] China	Electronic health record-based, machine-learning model to predict severe hypoglycemia leading to hospitalizations in older adults with diabetes	Cohort and modeling study/case-control design for a retrospective cohort.	ML predicts the risk of hypoglycemia in patients with diabetes	High
12. Tao et al. (2023) [47] China	Predicting three-month fasting blood glucose and glycated hemoglobin changes in patients with type 2 diabetes mellitus based on multiple machine learning algorithms	Prospective observational cohort study	XGBoost and ensemble learning prediction models for compliance with two blood glucose indicators in T2DM patients	High
13. Tarumi et al. (2021) [48] United States	Leveraging Artificial Intelligence to Improve Chronic Disease Care: Methods and Application to Pharmacotherapy Decision Support for Type-2 Diabetes Mellitus	A quantitative analysis method was validated	The TPGE model predicts HbA1c levels in patients with type 2 diabetes	High
14. Wang, S. et al. (2022) [49] China	Machine learning for the prediction of minor amputation in University of Texas grade 3 diabetic foot ulcers	Retrospective observational study	Machine learning XGBoost predicts minor amputations in diabetic foot ulcers (DFU3)	High
15. Yang, H. et al. (2022) [10] China	Predicting Risk of Hypoglycemia in Patients with Type 2 Diabetes by Electronic Health Record-Based Machine Learning: Development and Validation	Retrospective cohort study, Quantitative	The XGBoost machine learning model is used based on electronic health record (EHR) to predict hypoglycemia	High

**Table 2:** Themes.

Artificial Intelligence as a Tool for Blood Sugar Monitoring for Patients with Diabetes
Artificial Intelligence as a Decision Support for Diabetic Wounds and Complications
Patients’ Requests for Artificial Intelligence Capabilities in Relation to Tools

## Data Availability

Not applicable.

## Abbreviations

The following abbreviations are used in this manuscript:

AI	Artificial intelligence
XGBoost	Extreme Gradient Boosting
MMAT	Mixed Methods Appraisal Tool
ML	Machine Learning
REFS	Reverse Engineering and Forward Simulation
RF	Random forest
TPGE	Treatment Pathway Graph-Based Estimation
MLP	Multi-layer perception

## Appendix A

### Search Terms

**Search Action**	**CINAHL**	**ACM**	**Pubmed**
“Telemedicine” OR “Digital health” OR “Artificial Intelligence” OR “computational intelligence” OR “machine intelligence” (“artificial intelligence” [Title/Abstract] OR “computational intelligence” [Title/Abstract] OR “machine intelligence” [Title/Abstract])	36,812	161,607	254,611
“Diabetes mellitus” OR “Diabetes mellitus type 1” OR “diabetes mellitus type 2” OR “Diabetes” OR “type 2 diabetes” OR “diabetes type 2” OR “type 1 diabetes” OR “diabetes type 1”	258,392	5977	842,844
“Patient Centered Care” OR “Nurses Com-munity Health” OR Nurses OR “patient centered nursing” OR “patient focused care” OR “person centered care” OR “home health nurse” OR “nurse home health” OR “district nurse” OR “nursing staff” OR “Nursing” OR “nursing personnel” OR “nurses” OR “nurse s role” OR “nurses role”	970,454	13,763	492,126
#1 AND #2 AND #3	159	538	350
Filter: English language	104	538	212
Limitation 10 year (2014–2024)	51	460	216
Adult patients	51	460	114

## References

[1] Okafor C.N., Akosile C.O., Nkechi C.E., Okonkwo U.P., Nwankwo C.M., Okoronkwo I.L., Okpala P.U., Afonne A.J. (2023). Effect of educational intervention programme on the health-related quality of life (HRQOL) of individuals with type 2 diabetes mellitus in South-East, Nigeria. BMC Endocr. Disord..

[2] Dailah H.G. (2024). The Influence of Nurse-Led Interventions on Diseases Management in Patients with Diabetes Mellitus: A Narrative Review. Healthcare.

[3] Woldaregay A.Z., Årsand E., Walderhaug S., Albers D., Mamykina L., Botsis T., Hartvigsen G. (2019). Data-driven modeling and prediction of blood glucose dynamics: Machine learning applications in type 1 diabetes. Artif. Intell. Med..

[4] Tsalamandris S., Antonopoulos A.S., Oikonomou E., Papamikroulis G.A., Vogiatzi G., Papaioannou S., Deftereos S., Tousoulis D. (2019). The Role of Inflammation in Diabetes: Current Concepts and Future Perspectives. Eur. Cardiol..

[5] Vehi J., Mujahid O., Contreras I., Lidströmer N., Ashrafian H. (2022). Aim and Diabetes. Artificial Intelligence in Medicine.

[6] Kaze A.D., Santhanam P., Erqou S., Bertoni A.G., Ahima R.S., Echouffo-Tcheugui J.B. (2021). Microvascular disease and cardiovascular outcomes among individuals with type 2 diabetes. Diabetes Res. Clin. Pract..

[7] Khan R.M.M., Chua Z.J.Y., Tan J.C., Yang Y., Liao Z., Zhao Y. (2019). From Pre-Diabetes to Diabetes: Diagnosis, Treatments and Translational Research. Medicina.

[8] Cho M.K., Kim M.Y. (2021). Self-Management Nursing Intervention for Controlling Glucose among Diabetes: A Systematic Review and Meta-Analysis. Int. J. Environ. Res. Public Health.

[9] Deng P., Shi H., Pan X., Liang H., Wang S., Wu J., Zhang W., Huang F., Sun X., Zhu H. (2022). Worldwide Research Trends on Diabetic Foot Ulcers (2004–2020): Suggestions for Researchers. J. Diabetes Res..

[10] Yang H., Li J., Liu S., Yang X., Liu J. (2022). Predicting Risk of Hypoglycemia in Patients with Type 2 Diabetes by Electronic Health Record-Based Machine Learning: Development and Validation. JMIR Med. Inform..

[11] Yang L., Rong G.C., Wu Q.N. (2022). Diabetic foot ulcer: Challenges and future. World J. Diabetes.

[12] Habib S., Sangaraju S.L., Yepez D., Grandes X.A., Talanki Manjunatha R. (2022). The Nexus Between Diabetes and Depression: A Narrative Review. Cureus.

[13] Franquez R.T., de Souza I.M., Bergamaschi C.C. (2023). Interventions for depression and anxiety among people with diabetes mellitus: Review of systematic reviews. PLoS ONE.

[14] Skinner T.C., Joensen L., Parkin T. (2020). Twenty-five years of diabetes distress research. Diabet. Med..

[15] Bertachi A., Viñals C., Biagi L., Contreras I., Vehí J., Conget I., Giménez M. (2020). Prediction of Nocturnal Hypoglycemia in Adults with Type 1 Diabetes under Multiple Daily Injections Using Continuous Glucose Monitoring and Physical Activity Monitor. Sensors.

[16] Ansari R.M., Harris M.F., Hosseinzadeh H., Zwar N. (2023). Application of Artificial Intelligence in Assessing the Self-Management Practices of Patients with Type 2 Diabetes. Healthcare.

[17] Dankwa-Mullan I., Rivo M., Sepulveda M., Park Y., Snowdon J., Rhee K. (2019). Transforming Diabetes Care Through Artificial Intelligence: The Future Is Here. Popul. Health Manag..

[18] Ellahham S. (2020). Artificial Intelligence: The Future for Diabetes Care. Am. J. Med..

[19] Sridhar G., Lakshmi G. (2023). Ethical Issues of Artificial Intelligence in Diabetes Mellitus. Med. Res. Arch..

[20] Lag om egenvård. SFS 2022:1250. https://www.riksdagen.se/sv/dokument-och-lagar/dokument/svensk-forfattningssamling/lag-20221250-om-egenvard_sfs-2022-1250/.

[21] Riegel B., Jaarsma T., Strömberg A. (2012). A middle-range theory of self-care of chronic illness. ANS Adv. Nurs. Sci..

[22] Asmat K., Dhamani K., Gul R., Froelicher E.S. (2022). The effectiveness of patient-centered care vs. usual care in type 2 diabetes self-management: A systematic review and meta-analysis. Front. Public Health.

[23] Toobert D.J., Hampson S.E., Glasgow R.E. (2000). The summary of diabetes self-care activities measure: Results from 7 studies and a revised scale. Diabetes Care.

[24] Weinger K., Butler H.A., Welch G.W., La Greca A.M. (2005). Measuring diabetes self-care: A psychometric analysis of the Self-Care Inventory-Revised with adults. Diabetes Care.

[25] Ausili D., Barbaranelli C., Rossi E., Rebora P., Fabrizi D., Coghi C., Luciani M., Vellone E., Di Mauro S., Riegel B. (2017). Development and psychometric testing of a theory-based tool to measure self-care in diabetes patients: The Self-Care of Diabetes Inventory. BMC Endocr. Disord..

[26] Davies M.J., Aroda V.R., Collins B.S., Gabbay R.A., Green J., Maruthur N.M., Rosas S.E., Del Prato S., Mathieu C., Mingrone G. (2022). Management of Hyperglycemia in Type 2 Diabetes, 2022. A Consensus Report by the American Diabetes Association (ADA) and the European Association for the Study of Diabetes (EASD). Diabetes Care.

[27] Petersson L., Larsson I., Nygren J.M., Nilsen P., Neher M., Reed J.E., Tyskbo D., Svedberg P. (2022). Challenges to implementing artificial intelligence in healthcare: A qualitative interview study with healthcare leaders in Sweden. BMC Health Serv. Serv. Res..

[28] Gautier T., Ziegler L.B., Gerber M.S., Campos-Náñez E., Patek S.D. (2021). Artificial intelligence and diabetes technology: A review. Metabolism.

[29] Zhang J. (2022). Artificial Intelligence and Machine Learning Algorithm Optimization Applied in Health Big Data Digitization. Proceedings of the 2021 3rd International Conference on Artificial Intelligence and Advanced Manufacture.

[30] Lidströmer N., Aresu F., Ashrafian H., Lidströmer N., Ashrafian H. (2022). Introductory Approaches for Applying Artificial Intelligence in Clinical Medicine. Artificial Intelligence in Medicine.

[31] Topol E.J. (2019). Deep Medicine: How Artificial Intelligence Can Make Healthcare Human Again.

[32] Meetoo D.D., Ochieng B., Lidströmer N., Ashrafian H. (2022). AIM in Nursing Practice. Artificial Intelligence in Medicine.

[33] Whittemore R., Knafl K. (2005). The integrative review: Updated methodology. J. Adv. Nurs..

[34] Polit D.F., Beck C.T. (2021). Nursing Research: Generating and Assessing Evidence for Nursing Practice.

[35] Page M.J., McKenzie J.E., Bossuyt P.M., Boutron I., Hoffmann T.C., Mulrow C.D., Shamseer L., Tetzlaff J.M., Akl E.A., Brennan S.E. (2021). The PRISMA 2020 statement: An updated guideline for reporting systematic reviews. BMJ.

[36] Chan K.S., Chan Y.M., Tan A.H.M., Liang S., Cho Y.T., Hong Q., Yong E., Chong L.R.C., Zhang L., Tan G.W.L. (2022). Clinical validation of an artificial intelligence-enabled wound imaging mobile application in diabetic foot ulcers. Int. Wound J..

[37] Elhadd T., Mall R., Bashir M., Palotti J., Fernandez-Luque L., Farooq F., Mohanadi D.A., Dabbous Z., Malik R.A., Abou-Samra A.B. (2020). Artificial Intelligence (AI) based machine learning models predict glucose variability and hypoglycaemia risk in patients with type 2 diabetes on a multiple drug regimen who fast during ramadan (The PROFAST—IT Ramadan study). Diabetes Res. Clin. Pract..

[38] Hsu C.-C., Kao Y., Hsu C.-C., Chen C.-J., Hsu S.-L., Liu T.-L., Lin H.-J., Wang J.-J., Liu C.-F., Huang C.-C. (2023). Using artificial intelligence to predict adverse outcomes in emergency department patients with hyperglycemic crises in real time. BMC Endocr. Disord..

[39] James S., Armstrong M., Abdallah Z., O’Kane A.A. (2023). Chronic Care in a Life Transition: Challenges and Oppor-tunities for Artificial Intelligence to Support Young Adults with Type 1 Diabetes Moving to University. Proceedings of the 2023 CHI Conference on Human Factors in Computing Systems (CHI ‘23).

[40] Kozinetz R.M., Berikov V.B., Semenova J.F., Klimontov V.V. (2024). Machine Learning and Deep Learning Models for Nocturnal High- and Low-Glucose Prediction in Adults with Type 1 Diabetes. Diagnostics.

[41] Mert M., Vahabi A., Daştan A.E., Kuyucu A., Ünal Y.C., Tezgel O., Öztürk A.M., Taşbakan M., Aktuğlu K. (2024). Artificial intelligence’s suggestions for level of amputation in diabetic foot ulcers are highly correlated with those of clinicians, only with exception of hindfoot amputations. Int. Wound J..

[42] Mueller L., Berhanu P., Bouchard J., Alas V., Elder K., Thai N., Hitchcock C., Hadzi T., Khalil I., Miller-Wilson L.A. (2020). Application of Machine Learning Models to Evaluate Hypoglycemia Risk in Type 2 Diabetes. Diabetes Ther..

[43] Reddy R., Resalat N., Wilson L.M., Castle J.R., El Youssef J., Jacobs P.G. (2019). Prediction of Hypoglycemia During Aerobic Exercise in Adults with Type 1 Diabetes. J. Diabetes Sci. Technol..

[44] Robinson R., Liday C., Lee S., Williams I.C., Wright M., An S., Nguyen E. (2023). Artificial Intelligence in Health Care—Understanding Patient Information Needs and Designing Comprehensible Transparency: Qualitative Study. JMIR AI.

[45] Ruan Y., Bellot A., Moysova Z., Tan G.D., Lumb A., Davies J., van der Schaar M., Rea R. (2020). Predicting the Risk of Inpatient Hypoglycemia with Machine Learning Using Electronic Health Records. Diabetes Care.

[46] Shi M., Yang A., Lau E.S.H., Luk A.O.Y., Ma R.C.W., Kong A.P.S., Wong R.S.M., Chan J.C.M., Chan J.C.N., Chow E. (2024). A novel electronic health record-based, machine-learning model to predict severe hypoglycemia leading to hospitalizations in older adults with diabetes: A territory-wide cohort and modeling study. PLoS Med..

[47] Tao X., Jiang M., Liu Y., Hu Q., Zhu B., Hu J., Guo W., Wu X., Xiong Y., Shi X. (2023). Predicting three-month fasting blood glucose and glycated hemoglobin changes in patients with type 2 diabetes mellitus based on multiple machine learning algorithms. Sci. Rep..

[48] Tarumi S., Takeuchi W., Chalkidis G., Rodriguez-Loya S., Kuwata J., Flynn M., Turner K.M., Sakaguchi F.H., Weir C., Kramer H. (2021). Leveraging Artificial Intelligence to Improve Chronic Disease Care: Methods and Application to Pharmacotherapy Decision Support for Type-2 Diabetes Mellitus. Methods Inf. Med..

[49] Wang S., Wang J., Zhu M.X., Tan Q. (2022). Machine learning for the prediction of minor amputation in University of Texas grade 3 diabetic foot ulcers. PLoS ONE.

[50] Hong Q.N., Fàbregues S., Bartlett G., Boardman F., Cargo M., Dagenais P., Gagnon M.-P., Griffiths F., Nicolau B., O’Cathain A. (2018). The Mixed Methods Appraisal Tool (MMAT) version 2018 for information professionals and researchers. Educ. Inf..

[51] Europeiska Kommisionen (2019). Etiska Riktlinjer för Tillförlitlig AI. https://www.europarl.europa.eu/meetdocs/2014_2019/plmrep/COMMITTEES/JURI/DV/2019/11-06/Ethics-guidelines-AI_SV.pdf.

[52] Shi L., Fonseca V., Childs B. (2021). Economic burden of diabetes-related hypoglycemia on patients, payors, and employers. J. Diabetes Complicat..

[53] Kulzer B., Freckmann G., Ziegler R., Schnell O., Glatzer T., Heinemann L. (2024). Nocturnal Hypoglycemia in the Era of Continuous Glucose Monitoring. J. Diabetes Sci. Technol..

[54] Kodama S., Fujihara K., Shiozaki H., Horikawa C., Yamada M.H., Sato T., Yaguchi Y., Yamamoto M., Kitazawa M., Iwanaga M. (2021). Ability of Current Machine Learning Algorithms to Predict and Detect Hypoglycemia in Patients with Diabetes Mellitus: Meta-analysis. JMIR Diabetes.

[55] Sudharsan B., Peeples M., Shomali M. (2015). Hypoglycemia prediction using machine learning models for patients with type 2 diabetes. J. Diabetes Sci. Technol..

[56] Battelino T., Danne T., Bergenstal R.M., Amiel S.A., Beck R., Biester T., Bosi E., Buckingham B.A., Cefalu W.T., Close K.L. (2019). Clinical Targets for Continuous Glucose Monitoring Data Interpretation: Recommendations From the International Consensus on Time in Range. Diabetes Care.

[57] Vettoretti M., Cappon G., Facchinetti A., Sparacino G. (2020). Advanced Diabetes Management Using Artificial Intelligence and Continuous Glucose Monitoring Sensors. Sensors.

[58] McCormack B., McCance T.V. (2006). Development of a framework for person-centred nursing. J. Adv. Nurs..

[59] Lundin Gurné F., Jakobsson S., Lidén E., Björkman I. (2023). District nurses’ perspectives on health-promotive and disease-preventive work at primary health care centres: A qualitative study. Scand. J. Caring Sci..

[60] Guan Z., Li H., Liu R., Cai C., Liu Y., Li J., Wang X., Huang S., Wu L., Liu D. (2023). Artificial intelligence in diabetes management: Advancements, opportunities, and challenges. Cell Rep. Med..

[61] Norori N., Hu Q., Aellen F.M., Faraci F.D., Tzovara A. (2021). Addressing bias in big data and AI for health care: A call for open science. Patterns.

